# Fibromyalgia, gender, and employment: unveiling an overlooked reality in occupational health in Chile

**DOI:** 10.47626/1679-4435-2026-1527

**Published:** 2026-07-23

**Authors:** Margarita Zamora-Saa, Rodolfo Jara-Jegó

**Affiliations:** 1 Fundación Instituto Profesional Duoc UC, Santiago, Chile; 2 Department of Physical Medicine and Rehabilitation, Comprehensive Rehabilitation Center, Hospital San Camilo, San Felipe, Chile

**Keywords:** fibromyalgia, employment, occupational health, sex factors, psychosocial factors.

## Abstract

Fibromyalgia is a multifactorial syndrome characterized by chronic widespread pain, fatigue, and cognitive dysfunction that primarily affects women of working age. Because it lacks biological markers and its diagnosis is largely based on clinical criteria, fibromyalgia has been subject to gender biases that tend to minimize its symptoms and question the legitimacy of the condition. This delegitimization affects the employment trajectories of women with fibromyalgia, who experience higher rates of unemployment, withdrawal from employment, and discrimination. Studies indicate that only a minority are able to remain in the workforce and that many women feel compelled to conceal or minimize symptoms, which increases emotional exhaustion and worsens their health. In Chile, despite advances such as the enactment of Law 21,531 on fibromyalgia and non-cancer chronic pain, comprehensive programs integrating health and employment from a biopsychosocial perspective remain lacking. The aim of this article is to contribute to academic and social reflection on the occupational and psychosocial implications of fibromyalgia in women, highlighting the urgent need for inclusive strategies that support their ability to remain employed or return to work. Evidence indicates that employment plays a protective role by improving physical and mental well-being, providing economic independence, and fostering a sense of purpose. Therefore, the implementation of ergonomic interventions, reasonable accommodations, and psychoeducational strategies involving patients, families, health care teams, and employers should be prioritized. Only through inclusive policies with a gender perspective will it be possible to reduce occupational exclusion and improve the quality of life of individuals with fibromyalgia.

## INTRODUCTION

Fibromyalgia (FM) is a disorder of complex, multifactorial etiology and pathogenesis, whose prevalence is significantly higher among women of working age [^1^]. In 1990, the American College of Rheumatology defined FM as a condition characterized by a history of widespread pain for at least 3 months, along with tenderness on palpation at 11 of 18 tender points. Since then, the diagnostic criteria have evolved substantially. The 2010 and 2011 revisions, and particularly the 2016 revision, removed tender points as a diagnostic requirement and emphasized the presence of generalized pain in at least four of five body regions, together with associated symptoms such as fatigue and cognitive dysfunction. Currently, the diagnosis remains clinical, and chronic widespread pain continues to be a central diagnostic criterion [^2^].

This article highlights the urgent need to further investigate the occupational challenges faced by individuals with FM in Chile, particularly women. It seeks to encourage reflection and critical analysis regarding the need for a biopsychosocial approach to the management of FM, with the aim of fostering discussion in both academic and institutional settings. The ultimate goal is to support the development of strategies that promote the labor market inclusion and workforce reintegration of women diagnosed with this condition. Despite advances in the clinical understanding of FM, its impact extends beyond the biological dimension. Women living with this condition encounter a complex network of social and cultural barriers that transcend medical issues and are particularly evident in the workplace. It is precisely at the intersection between health, gender, and employment that the psychosocial risks contributing to the exclusion and discrimination of women with FM become more apparent.

## SEARCH STRATEGY

A narrative review of the scientific literature was conducted to support the reflections presented in this article. PubMed was the primary database consulted, supplemented by SciELO and Google Scholar, and only articles published between 2019 and 2025 were considered. The search strategy included the terms “fibromyalgia,” “employment,” “work disability,” “gender,” “occupational health,” and “psychosocial factors,” combined using Boolean operators. Original studies, reviews, qualitative analysis, and policy documents published in English, Spanish, or Portuguese were included, with priority given to those addressing the occupational impact of FM, gender inequalities, and associated psychosocial risks. References were selected based on their thematic relevance and conceptual contribution to the proposed analysis; therefore, this study does not constitute a systematic review. The reviewed evidence is summarized in Table 1.

**Table 1:** Occupational impact of fibromyalgia: selected studies

Ref.	Author/year	Country/context	Design/type	Main results
1	Cordeiro et al., 2023	Brazil/occupational health	Opinion article	Treatment of FM and occupational physician’s role: chronic pain impairs productivity; greater expertise in occupational medicine in urgently needed
3	Tenti et al., 2024	Italy/working with FM	Qualitative (surveys and interviews)	Workplace accommodations and work flexibility reduce workforce withdrawal; significant impact on job retention
4	Duhn et al., 2021	Denmark/chronic pain	Cohort study	One-third of women with FM are outside the labor force
5	Olivia-Moreno & Vilaplana-Prieto, 2024	Spain/costs associated with FM	Economic analysis (survey)	High costs from labor market exclusion (employment rate: 21.9% among women with FM vs. 64.8% in the general population)
6	Chen et al., 2019	International/occupational	Conceptual synthesis	Limited research on the occupational impact of FM worldwide; need for workplace accommodations
7	Mukhida et al., 2020	Canada/occupational	Scoping review	Occupational challenges, symptom concealment, and experiences of stigma
8	Zitko et al., 2021	Chile/chronic pain	Prevalence analysis	Second most common musculoskeletal disorder among women, significant impact on quality of life
9	Espinoza et al., 2022	Chile/chronic pain	Cost analysis	FM is associated with a substantial economic burden and a high prevalence of depression
10	Mohabbat et al., 2023	United States/occupational health	Cross-sectional study	Type of occupation is associated with symptom severity and functional limitations
11	Laroche et al., 2019	France/survey (n = 955)	Cross-sectional study	Lack of recognition of the condition by coworkers and supervisors is associated with greater symptom severity and work absenteeism

Ref. = reference number; FM = fibromyalgia.

## PREVALENCE AND SEX DISTRIBUTION

FM is the third most common musculoskeletal disorder, and its prevalence increases with age. Globally, its prevalence is estimated to range between 2% and 3% [^12^]. A prevalence of 2.45% was found in an adult Spanish sample, similar to that observed in Europe as a whole (2.64%) [^13^]. In Brazil, approximately 2% of the population has been diagnosed with FM [^14^]. The syndrome predominantly affects women of working age [^1^,^3^], with a female-to-male prevalence ratio of 9:1 [^15^].

## OCCUPATIONAL IMPACT OF FIBROMYALGIA

The onset of pain associated with FM occurs predominantly during working age [^3^]. Chronic widespread pain among workers with FM has been shown to impose an economic burden on health care systems, which increases as the condition progressively limits functional capacity and reduces work productivity through impaired performance [^1^,^4^]. Consequently, FM is associated with difficulties maintaining employment, lower levels of social participation, and substantial direct and indirect costs related to sick leave and disability pensions. Job loss and early retirement due to disability are directly related to the overall disease burden attributable to FM [^4^].

Employment and unemployment rates among individuals with FM vary according to the type of study. Some studies found that between 35% and 50% of individuals with FM are not employed, whereas others report figures ranging from 10% to 57% [^3^] (see reference 6 and the studies cited therein). Approximately 30% of women diagnosed with FM are estimated to lose their jobs because of frequent work absences [^16^]. Regarding employment rates among women with FM, Duhn et al., found that only one-third participate in labor force [^4^]. A Spanish study based on national registry and survey data showed that, in 2021, only 21.9% of women diagnosed with FM were employed, compared with 64.8% of women in the general population who held paid employment during the same year. The study highlighted that employment rates among women with FM may be substantially lower than those observed in the general population, reflecting the specific challenges faced by this group in maintaining employment [^5^].

## RESEARCH GAPS IN FIBROMYALGIA AND WORK

In recent years, scientific interest in the etiology of FM has increased. However, relatively few studies have examined the occupational consequences of the disease in depth from a biopsychosocial perspective. A search conducted in PubMed, one of the most important biomedical databases, identified more than 15,441 publications related to FM, of which slightly more than one-third were published between 2019 and 2025, reflecting growing interest in this condition. Nevertheless, Chen et al. highlighted the scarcity of literature addressing the occupational impacts of FM worldwide [^6^]. In South America, Brazil leads scientific production on the subject and ranks third globally, behind the United States and Spain [^17^].

## FIBROMYALGIA, GENDER, AND LABOR MARKER EXCLUSION: THE ROLE OF PSYCHOSOCIAL RISK

Psychosocial risk constitutes a critical factor contributing to the progressive deterioration of health among women with FM and, in many cases, to withdrawal from paid employment. These women are often compelled to conceal or minimize their symptoms in order to meet workplace demands, which increases feelings of guilt, frustration, and emotional exhaustion. In unsupportive work environments lacking inclusive policies and workplace accommodations, they are frequently perceived as less reliable or less productive, making them vulnerable to discrimination and exclusion. This dynamic directly affects their self-esteem, motivation, and ability to remain employed [^7^].

Women with FM face substantial emotional demands in both the workplace and the domestic sphere. This situation is exacerbated by the invisibility of symptoms, limited medical and social validation, and the diagnostic ambiguity surrounding the condition [^18^]. Unlike other diseases with clearly identifiable biological markers, FM is diagnosed primarily through clinical criteria based on subjective symptoms, such as pain, fatigue, sleep disturbances, and cognitive dysfunction [^12^]. The absence of conclusive diagnostic tests had created uncertainty for both health care professionals and patients themselves, contributing to the characterization of FM as a “questionable” or “psychosomatic” condition. Because this disease predominantly affects women, it has been associated with stereotypes such as emotional fragility or hysteria, reinforcing the perception that it is “less legitimate” than conditions with objective clinical signs. This gender bias in diagnosis and health care represents a form of structural violence that perpetuates inequalities in access to health care and decent work.

The discrimination against women with FM is multidimensional and affects their medical, occupational, and social trajectories. Recent studies show that these women experience a form of double invisibility: first, because of the challenges associated with obtaining a clinical diagnosis; and second, because they belong to a group that has historically been undervalued within biomedical systems, where chronic pain in women is often minimized or interpreted exclusively from a psychological perspective [^19^]. In the workplace, this medical delegitimization translates into a range of discriminatory practices, including denial of sick leave, lack of workplace accommodations, and the stigma of being perceived as “less productive,” which may lead to isolation, exclusion from meaningful work activities, and even forced loss job [^20^].

Health care professionals frequently express negative judgments, questioning the legitimacy of patient’s symptoms or attributing them solely to emotional factors, thereby creating barriers to timely, dignified, and person-centered care [^19^]. This systematic invalidation affects the self-esteem, quality of life, and identity of women with FM, reinforcing forms of symbolic violence that intersect with social conditions such as poverty, domestic overload, and limited access to support networks.

Within this context, discrimination against women with FM should be understood as a structural phenomenon sustained by gender inequalities in medicine and the workplace, rather than a series of isolated incidents of unfair treatment. Recent research has proposed addressing this issue through comprehensive strategies that include raising awareness among health care professionals, incorporating gender equity principles into workplace policies, and developing support systems that recognize the legitimacy of patients’ suffering and the need for reasonable accommodations [^18^].

To assess the magnitude of the problem in Chile and inform potential local solutions that take psychosocial risks and gender-related barriers into account, the following sections reviews the national available evidence to better understand the health and economic impact of FM, as well as the institutional responses implemented in recent years.

## NATIONAL EVIDENCE ON FIBROMYALGIA: PREVALENCE, COSTS, AND PUBLIC POLICIES

In Chile, investigation on FM is still limited. According to Zitko et al., using data from the National Health Survey, FM has an estimated prevalence of 4% [^8^] and a significant impact on patients’ well-being, as measured by reductions in health-related quality of life. FM is also the second most common specific musculoskeletal disorder, particularly among women, after chronic low back pain [^8^].

With regard to associated costs, Espinoza et al. estimated that the annual expenditure attributable to chronic-pain related conditions amounts to USD 943,413,490, of which 80.9% corresponded to the five diseases studied (knee osteoarthritis, hip osteoarthritis, low back pain, shoulder disorders, and FM), with low back pain and FM accounting for the greatest economic burden on the health care system. In particular, FM was associated with the highest costs, largely due to comorbid depression, reaching approximately USD 9.2 million annually [^9^].

Furthermore, Ruggeri-Raglianti et al., using data from the Department of Health Statistics and Information and the National Institute of Statistics, analyzed hospital discharge rates for FM in Chile between 2019 and 2022. An overall hospital discharge rate of 0.27 was observed, with higher rates among women (0.47) than men (0.05). The 40-49 year age group had the highest rate (0.69), and the mean length of hospital stay was 5.25 days [^21^].

In terms of public policies, in 2016 the Chilean Ministry of Health developed a technical guidance document intended to support the clinical management of FM. This document provides health care professionals with recommendations for the detection, referral, medical treatment, and rehabilitation of individuals diagnosed with FM. Subsequently, in 2023, Law N.º 21,531 on FM and non-cancer chronic pain was enacted, promoting and guaranteeing comprehensive health care with the aim of improving the quality of health of individuals affected by these diseases [^22^]. In this context, a key measure to improve patient well-being would be the incorporation of therapeutic programs designed to prevent withdrawal from employment and/or facilitate return to work. Nevertheless, to date, there is no specific program or protocol that comprehensively addresses the psychosocial and occupational problems associated with FM.

## FIBROMYALGIA AND EMPLOYMENT: AN OVERLOOKED REALITY IN CHILE

Despite regulatory advances and the available evidence regarding the burden of FM in Chile, the employment reality of individuals living with this condition remains largely overlooked. The data presented highlight the magnitude of the problem in terms of health and economy; however, little is known about the day-to-day experiences of those affected, particularly with respect to employment.

To illustrate this issue, between 2021 and 2024 21 women diagnosed with FM were treated at a specific unit within a Chilean public hospital. Of these, 16 were of working age (39-63 years), and 14 left their jobs or substantially reduced their working hours and workload. On average, this change occurred 5.27 years after diagnosis. This decision was likely influenced by both psychosocial and emotional distress and the progressive decline in functional capacity. Such situation is neither isolated nor unique to the Chilean context. Recent studies have shown that FM is a frequent cause of withdrawal from employment, reduced income, and difficulties maintaining stable employment, particularly among women [^6^]. This preliminary report, based on the experience of a rehabilitation service within a public hospital, represents an initial warning with regard to the magnitude of the employment-related challenges associated with FM in women. Given this reality, there is a need to better understand labor force participation rates among Chilean women with FM. However, to date, no national studies have examined this issue, and no data are available regarding employment, formal dismissals, negotiated resignations, or voluntary withdrawal from employment associated with the disease.

The Chilean public health system faces structural limitations in comprehensively addressing the employment-related challenges associated with FM. At present, there are no institutionalized support, counselling, or mediation programs that simultaneously consider the needs of female workers with FM and those of their employers. Clear government guidelines are required to enable the implementation of early intervention processes aimed at preventing withdrawal from employment before it becomes irreversible. One of the few national studies to highlight the need to promote return to work while taking into account the functional status of individuals with chronic pain (or, when appropriate, to facilitate recognition of potential disability) is that of Ramírez-Robles et al.; however, the study does not provide specific statistical data on the employment situation these patients [^23^].

Most health care services within the public health system focus exclusively on the treatment of physical and psychological symptoms, without effective coordination with the employment sector. This omission may reflect both a lack of awareness among clinical teams regarding the benefits of employment in the management of FM and logistical or regulatory barriers to establishing effective links with workplaces and employers. As noted by Sanjuan-Sánchez et al., health care professionals should possess the competencies necessary to identify factors that may exacerbate symptoms and to promote modifications to working conditions. Such measures would provide direct support for individuals with FM to remain in the workforce [^24^].

## THE PROTECTIVE ROLE OF EMPLOYMENT AMONG WOMEN WITH FIBROMYALGIA

Remaining in the workforce is essential for women with FM, as employment may help prevent further deterioration of clinical condition. Numerous studies have documented the benefits of continuing to work despite the disease [^3^,^4^]. Nevartheless, factors such as the degree of functional impairment, the level of disability, and symptom severity directly influence work ability and affect the ability to remain employed.

A qualitative study on quality of life among women with FM found that those who are able to maintain employment, particularly when working conditions are adapted to their health needs, experience better mental health, fewer depressive and anxiety symptoms, and a higher quality of life. These benefits appear to result, in part, from the social support and personal fulfillment associated with maintaining employment. In contrast, unemployment and job loss were associated with substantial emotional and social deterioration [^25^]. Similarly, Mohabbat et al. highlighted that numerous studies have shown that employment is related to improved physical health, mental health, and overall well-being, to the extent that it may be considered a therapeutic intervention for individuals with FM [^10^].

Furthermore, maintaining employment reduces the risk of poverty and financial dependence. Salaffi et al. emphasize that work constitutes a central component of life for many individuals with FM, as it strengthens self-esteem, provides a sense of purpose, and promotes economic independence [^26^]. Individuals with FM often face additional treatment-related expenses and difficulties accessing social benefits; therefore, maintaining employment facilitates more positive coping with the disease and reinforces feelings of usefulness and productivity. In addition, both reduced labor force participation and barriers to obtaining formal recognition of disability impose a substantial economic burden on the families of women with FM, placing this group in a state of vulnerability that remains insufficiently recognized in social and political spheres [^24^].

Taken together, this evidence reinforces the need to implement support measures and workplace accommodations targeted at women with FM, with the aim of preventing their premature exclusion from the labor market and maximizing the benefits that employment can provide, even in the context of a chronic illness.

## WORKPLACE INTERVENTIONS FOR THE MANAGEMENT OF FIBROMYALGIA

Understanding the employment trajectories of women with FM, both past and present, is essential for designing comprehensive support programs that take into account the physical, emotional, and organizational demands related to their multiple roles. Evidence shows that individuals with FM have a reduced tolerance for fatigue, which limits their ability to adapt to work environments characterized by high physical, psychosocial, and organizational demands.

A study conducted in France involving 955 employed women diagnosed with FM observed that the main risk factors associated with sick leave were not related to the severity of clinical symptoms or individual characteristics, but rather to workplace factors, such as lack of recognition of the disease, organizational demands, absence of workplace accommodations, and commuting distance [^11^]. These findings reinforce the need to evaluate jobs from both ergonomic and psychosocial perspectives in order to implement reasonable accommodations that support the continued labor force participation of women with FM.

Among the recommended measures is the redesign of job duties to avoid strenuous physical activities such as lifting loads, frequent bending, prolonged postures, and repetitive movements, all of which may exacerbate symptoms [^27^], particularly in women [^28^]. Moreover, Tenti et al. emphasize the importance of interventions aimed at adapting the workplace through educational strategies directed at workers, employers, and work teams alike [^3^]. These measures help create more inclusive work environments, promoting employment sustainability and preventing withdrawal from employment.

However, interventions should not be limited to physical modifications of the workplace. Explicit recognition of FM as a legitimate condition by coworkers and supervisors may have an impact that is as important as, or even greater than, conventional clinical treatment [^11^]. Evidence supports that workplace support plays a decisive role in both continued employment and return to work among individuals with FM. Dépelteau et al., reported that support from coworkers and supervisors is a key factor influencing the decision to return to work following periods of work disability [^29^]. Similarly, a qualitative study found that women who left their jobs perceived less support from coworkers and the broader work environment than those who remained employed [^7^]. Likewise, Ganesh & Lazar, drawing on testimonies from individuals with FM, observed that disclosure of the diagnosis, when met with understanding and empathy, can facilitate the collaborative redistribution of workload and foster supportive dynamics within work teams [^30^].

In this regard, the implementation of multidimensional psychoeducational strategies that include the affected individual, their family, and their socio-occupational environment constitutes a key role for strengthening employment retention and reducing psychosocial vulnerability. Furthermore, greater workplace flexibility is recommended with respect to working hours, workload intensity, and even work location [^31^]. For example, opportunities for remote work could be offered, even if this may entail a potential reduction in income. As discussed previously, substantial evidence indicates that remaining in the workforce promotes the well-being of individuals with FM; therefore, every reasonable effort should be made to prevent job loss.

## CONCLUSIVE REMARKS

FM is not only a clinical challenge but also a major socioeconomic problem, closely related to job loss, prolonged sick leave, disability pension claims, and substantial health care costs for public health systems and affected families [^1^]. In light of this reality, there is an urgent need for clear government guidelines that promote early support based on a biopsychosocial approach, with the aim of preventing withdrawal from employment before it becomes irreversible.

An effective approach requires the simultaneous assessment of ergonomic and psychosocial risks present in the workplace. From an ergonomic perspective, it is essential to identify and modify tasks involving physical demands, repetitive movements, or prolonged postures, as these conditions may exacerbate symptoms [^27^]. From a psychosocial perspective, factors such as emotional demands, experiences of discrimination, the quality of interpersonal relationships with coworkers and supervisors, and the socioeconomic vulnerability of individuals with FM. When integrated within a biopsychosocial framework, these elements can provide a more comprehensive understanding of the impact of the disease and inform the design of more effective interventions. It would also be important to conduct qualitative studies exploring coworkers’ perspectives regarding potential additional workload, work overload, and the satisfaction or dissatisfaction associated with assuming tasks previously performed by individuals with FM. This line of research would allow for a more comprehensive understanding of the organizational impact of the condition and support the development of interventions that benefit both workers with FM and their work teams.

Furthermore, it is essential to strengthen the training of health care professionals through a comprehensive approach that recognizes FM as a legitimate and potentially disabling medical condition. This training should incorporate a gender perspective as a cross-cutting component, given its importance in challenging the historical stigmas and biases that have rendered chronic pain in women invisible.

Finally, se FM must be given greater institutional and legislative visibility, ensuring the effective exercise of labor rights, access to adequate social benefits, and timely coverage of treatment. Only through a biopsychosocial approach will it be possible to advance meaningful labor market inclusion, improve the quality of life of affected individuals, and reduce the impact of FM on society.

## Data Availability

The data supporting the findings of this study are available within the article.
